# The effect of orofacial complete Freund’s adjuvant treatment on the expression of migraine-related molecules

**DOI:** 10.1186/s10194-019-0999-7

**Published:** 2019-04-29

**Authors:** Tamás Körtési, Bernadett Tuka, Aliz Nyári, László Vécsei, János Tajti

**Affiliations:** 1 Department of Neurology, Faculty of Medicine, Albert Szent-Györgyi Clinical Center University of Szeged, Semmelweis u. 6, Szeged, H-6725 Hungary; 2 MTA-SZTE Neuroscience Research Group, University of Szeged, Semmelweis u. 6, Szeged, H 6725 Hungary; 3 Department of Neurology, Interdisciplinary Excellence Centre, Faculty of Medicine, Albert Szent-Györgyi Clinical Center, University of Szeged, Semmelweis u. 6, Szeged, H-6725 Hungary

**Keywords:** Migraine, Trigeminovascular system, CFA, CGRP, preproPACAP

## Abstract

**Background:**

Migraine is a neurovascular primary headache disorder, which causes significant socioeconomic problems worldwide. The pathomechanism of disease is enigmatic, but activation of the trigeminovascular system (TS) appears to be essential during the attack. Migraine research of recent years has focused on neuropeptides, such as calcitonin gene-related peptide (CGRP) and pituitary adenylate cyclase-activating polypeptide 1–38 (PACAP1–38) as potential pathogenic factors and possible therapeutic offensives. The goal of present study was to investigate the simultaneous expression of CGRP and precursor of PACAP1–38 (preproPACAP) in the central region of the TS in a time-dependent manner following TS activation in rats.

**Methods:**

The right whisker pad of rats was injected with 50 μl Complete Freund’s Adjuvant (CFA) or saline. A mechanical allodynia test was performed with von Frey filaments before and after treatment. Transcardial perfusion of the animals was initiated 24, 48, 72 and 120 h after injection, followed by the dissection of the nucleus trigeminus caudalis (TNC). After preparation, the samples were stored at − 80 °C until further use. The relative optical density of CGRP and preproPACAP was analyzed by Western blot. One-way ANOVA and Kruskal-Wallis followed by Tukey post hoc test were used to evaluate the data. Regression analysis was applied to explore the correlation between neuropeptides expression and hyperalgesia.

**Results:**

Orofacial CFA injection resulted in significant CGRP and preproPACAP release in the TNC 24, 48, 72 and 120 h after the treatment. The level of neuropeptides reached its maximum at 72 h after CFA injection, corresponding to the peak of facial allodynia. Negative, linear correlation was detected between the expression level of neuropeptides and value of mechanonociceptive threshold.

**Conclusion:**

This is the first study which suggests that the expression of CGRP and preproPACAP simultaneously increases in the central region of activated TS and it influences the formation of mechanical hyperalgesia. Our results contribute to a better understanding of migraine pathogenesis and thereby to the development of more effective therapeutic approaches.

## Background

Recently, two peptides of key molecules have been highlighted for their involvement in the pathomechanism of primary headache disorders: calcitonin gene-related peptide (CGRP), as an “old warrior” and pituitary adenylate cyclase-activating polypeptide 1–38 (PACAP1–38), which is a newer, potential target for headache therapies. These peptides are very similar in features and functions: e.g. they are potent vasodilators, they are present in both the peripheral and central nervous system and they can function in the transmission of nociception and neurogenic inflammation. Subsequently, they have gained ground in the therapeutic developments in migraine. In 2004, the first CGRP receptor antagonists (gepants) effectively terminated migraine in humans, but nowadays several other anti-CGRP treatments are in clinical trials or are under development (anti-CGRP and anti-CGRP receptor monoclonal antibodies) for the prevention of migraine [1]. PACAP1–38 also has pivotal role in migraine, as indicated by several preclinical [2–4] and clinical [5–9] examinations, but there are fewer confirmed results targeted on the PACAP1–38 antibody therapies [10]. The analogous behaviour of these peptides presents the possibility that anti-PACAP1–38 treatments could provide a therapeutic advantage for migraineurs who do not respond the anti-CGRP therapies.

Thus it is interesting to investigate the simultaneous release of CGRP and precursor of PACAP1–38 (preproPACAP) in a migraine-related environment. In our case, the orofacial Complete Freund’s Adjuvant (CFA) rat model was selected to determine these alterations. CFA has been used for animal modelling of autoimmune and inflammatory illnesses for nearly 50 years and it is an accepted model of orofacial inflammatory hyperalgesia [11–16].

The precise mode of action of CFA is not known, but it primarily triggers an inflammatory reaction through the activation of the cellular immune response [11]. The CFA-induced chemical stimulation of the orofacial area can activate the extra- and intracranial trigeminal primary afferents, which provide the sensory innervation of the face and oral cavity, as well as the vasculature-associated meningeal nociceptive afferents. The cell bodies of these peripheral fibres are located in the trigeminal ganglion (TRG), while the central projections of these neurons terminate in the trigeminal nucleus caudalis (TNC). Sensitisation of the peripheral fibres and the second order nociceptive neurons in the TNC can contribute to the phenomenon of facial allodynia developing in primary headache disorders [17]. Perception of pain can be connected to the thalamic third order neurons [18–22].

This commonly used peripheral inflammation model can integrate those mechanisms which are involved in migraine, because several primary headache diseases associated with inflammation of extracranial structures, such as the temporomandibular joint (TMJ) or the sinuses, can be evoked by similar mechanisms to those mentioned above [16, 23, 24]. Comorbidity was also observed between migraine and TMJ disorders [25], therefore orofacial inflammation induced by CFA might be suitable to generate hyperalgesia/allodynia on the face by the activation/sensitization of the trigeminovascular system (TS), namely to mimic the features of migraine [12].

In the course of our research we aimed to model allodynia-associated activated TS using CFA in rats. We used von Frey filaments to assess nociceptive sensitization and measure the temporal changes in facial mechano-nociceptive threshold after periods of 24, 48, 72, and 120 h following CFA injection. In order to estimate the release of neuropeptides in the TNC in the assessed time periods following CFA injection, Western blot assay was used with specific preproPACAP and CGRP antibodies.

This the first study which evaluated the presence of preproPACAP and CGRP together in the TNC and correlated the behavioural changes following facial inflammation. These investigations might provide better insight into the background of trigeminal pain disorders.

## Materials and methods

### Animals

Thirty young adult (10–12 weeks old) male Sprague-Dawley rats were used for the experiments. The animals were bred and maintained under standard laboratory conditions with a 12–12-h light/dark cycle at 24 ± 1 °C and approx. 50% relative humidity in the Laboratory Animal House of the Department of Neurology. The rats had free access to standard rat chow and water.

### Ethics

All experimental procedures performed in this study complied fully with the guidelines of Act 1998/XXVIII of the Hungarian Parliament on Animal Experiments (243/1988) and with the recommendations of the International Association for the Study of Pain and European Communities Council (86/609/ECC). The studies were in harmony with the Ethical Codex of Animal Experiments and were approved by the Ethics Committee of the Faculty of Medicine, University of Szeged, XI./1102/2018.

### Drugs

Complete Freund’s Adjuvant (killed mycobacteria suspended in paraffin oil; 1 mg/ml) was obtained from Sigma-Aldrich Corporation (St. Louis, MO, USA), and 50 μl was administered per animal.

### Orofacial pain sensitivity tested with von Frey filaments

In this experiment mechanical pain thresholds of the orofacial region were determined with von Frey filaments. Tests were performed before (0) and at 24, 48, 72, 120 h after CFA/saline injection. Animals were lightly restrained using a soft cotton glove in order to allow an easier habituation, then a set of calibrated nylon monofilaments (SENSElab – AESTHESIOMETER, SOMEDIC Sales AB, Box 194, 242 31 Hörby) was used with increasing strengths (0.39–8.3 g) to measure facial mechanosensitivity. Filaments were applied in ascending order, starting from the 3.3 g filament during control measurements and the 0.39 g filament after CFA treatment. The mechanonociceptive threshold was defined as the lowest force evoking at least two withdrawal responses (face stroking with the forepaw or head shaking) out of five stimulations [12].

### Experimental protocol

In our experiment five animal groups were created: 1 control group and 4 groups with CFA treatment. The groups and treatments are presented in Table 1.

**Table 1:** Experimental groups

Group	Control (*n* = 6)	CFA 1. (*n* = 6)	CFA 2. (*n* = 6)	CFA 3. (*n* = 6)	CFA 4. (*n* = 6)
Orofacial treatment	50 μl saline	50 μl CFA	50 μl CFA	50 μl CFA	50 μl CFA
Perfusion and dissection	24 h after treatment	24 h after treatment	48 h after treatment	72 h after treatment	120 h after treatment

First, the rats were anesthetized with intraperitoneal 4% chloral hydrate solution (10 ml/kg bw dose) and the anesthesia was maintained throughout the experiment. Next, 50 μl of CFA was injected into the right whisker pad. Control rats were injected with an equal volume of saline. The von Frey allodynia test was performed before and after treatment, as described above. Transcardial perfusion of the animals with 200 ml phosphate-buffered saline (PBS) was initiated 24, 48, 72 and 120 h after injection. The medullary segment containing TNC (between rostral 1 mm and caudal 5 mm from the obex) was removed. After preparation, the samples were stored at − 80 °C until further use. The relative optical density of CGRP and preproPACAP was analyzed by Western blot.

### Protein quantification by Western blot analysis

The samples were sonicated in ice-cold buffer containing 50 mM Tris-HCl, 150 mM NaCl, 0.1% Igepal. 0.1% cholic acid, 2 mg/ml leupeptin, 2 mM phenylmethylsulphonyl fluoride, 1 mg/ml pepstatin, 2 mM ethylenediaminetetraacetic acid (EDTA), and 0.1% sodium dodecyl sulfate (SDS). After homogenization, the samples were centrifuged at 12.000 rpm for 10 min at 4 °C, and supernatants were aliquoted and stored at 20 °C until use. The protein concentration was determined by the BCA Protein Assay Kit using BSA as the standard. Prior to protein separation, each sample was mixed with the sample buffer, and denatured by boiling for 5 min. Equal amounts of protein samples (20 mg/lane) were separated by standard SDS polyacrylamide gel electrophoresis on 10% Tris-Glycine gel and electrotransferred onto an AmershamHybond-ECL nitrocellulose membrane (0.45-mm pore size). We used the Page Ruler Prestained Protein Ladder (10–170 kDa) to define approximate molecular weights. Following blotting, the membranes were blocked for 1 h at room temperature in Tris-buffered saline containing Tween 20 (TBST) and 5% nonfat dry milk powder. Subsequently, the membranes were incubated in TBST containing 1% nonfat dry milk and anti-PACAP antibody (against the C terminal; ab174982, dilution: 1:500, incubation parameters: overnight, 4 °C) or anti-CGRP antibody (Sigma-Aldrich-c8198, dilution: 1:2000, incubation parameters: overnight, room temperature), anti-glyceraldehyde 3-phosphate dehydrogenase (GAPDH) antibody (D16H11, dilution: 1:1000, incubation parameters: overnight, room temperature). The following day, the membranes were incubated in TBST containing 1% nonfat dry milk powder and horseradish peroxidase-conjugated goat anti-rabbit secondary antibody (sc-2030, Santa Cruz Biotechnology) for 2 h at room temperature. The protein bands were revealed with Syngene PXi 6 Access Touch Gel Documentation System.

### Statistical analysis

The Shapiro-Wilk test was used to determine the distribution of data. Data of the Western blot analysis followed a normal distribution, so after the One-way ANOVA test we used the Tukey’s post hoc test to analyze the results. The data from the von Frey allodynia test did not show a normal distribution, therefore we used the Kruskal-Wallis test with Tukey’s post hoc test. A probability level of *p* < 0.05 was considered significant. Mean ± SD are represented in the diagrams. Regression analyses were performed between the levels of neuropeptides and the value of hyperalgesia with SPSS 20.0 Software.

## Results

### Orofacial CFA treatment resulted in significant preproPACAP increase in the TNC

**Fig. 1:**
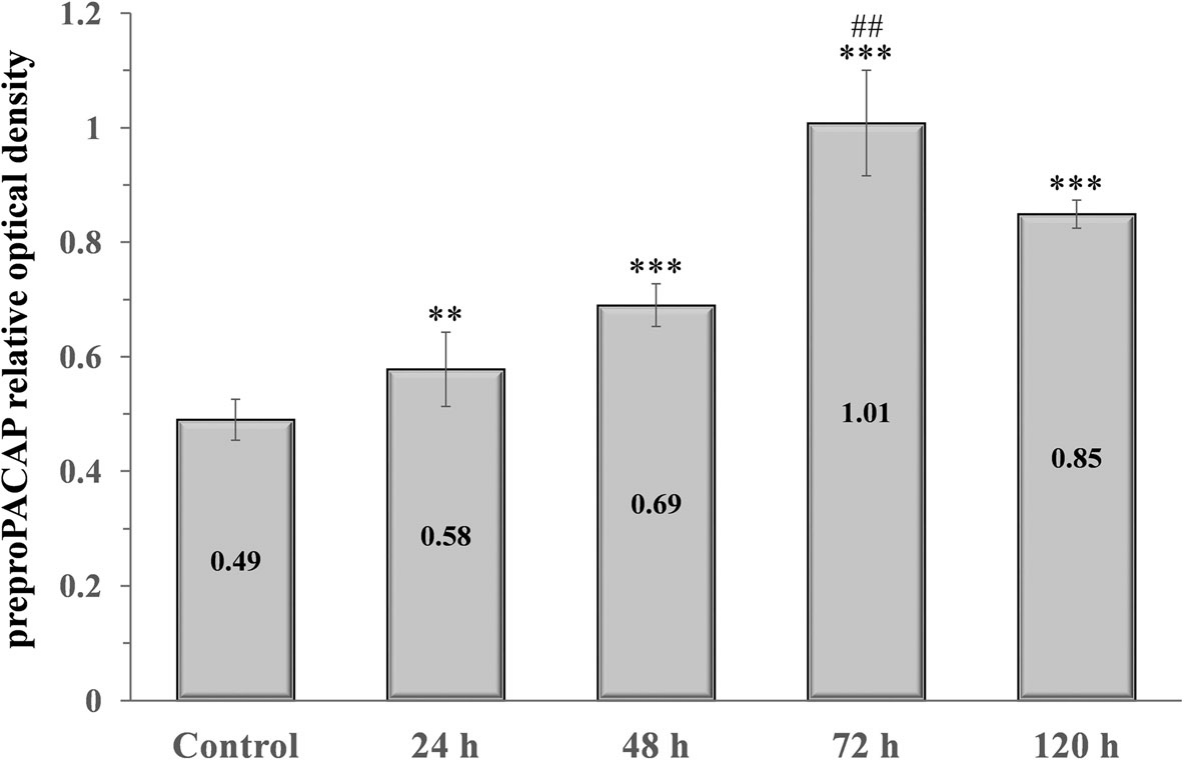
Relative optical density of the preproPACAP protein in the TNC following orofacial CFA treatment. *** *p* < 0.001 vs. Control group, ** *p* < 0.01 vs. Control group, ## *p* < 0.01 vs. 120 h group, Mean ± SD, *n* = 6

**Fig. 2:**
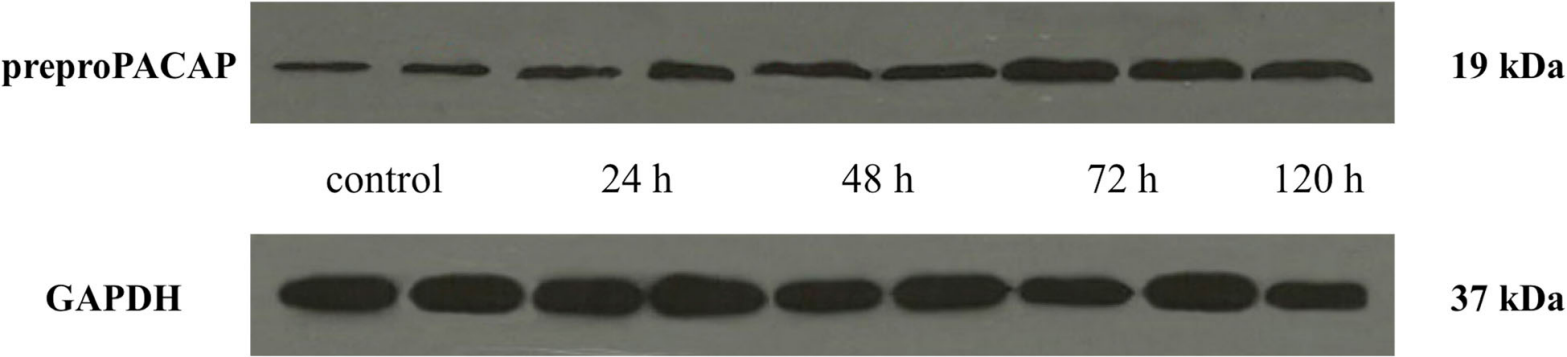
Western blot of preproPACAP and GAPDH expression in the TNC

A significant increase in the relative optical density of preproPACAP was observed 24 (0.58), 48 (0.69), 72 (1.01) and 120 (0.85) hours after CFA treatment compared to the control group (0.49). The highest preproPACAP concentration was measured 72 h after the CFA treatment. The difference between the 72 h and 120 h groups was significant, therefore we did not feel it necessary to include other group investigations (Figs. 1 and 2).

### Orofacial CFA treatment significantly elevated CGRP relative optical density in the TNC

**Fig. 3:**
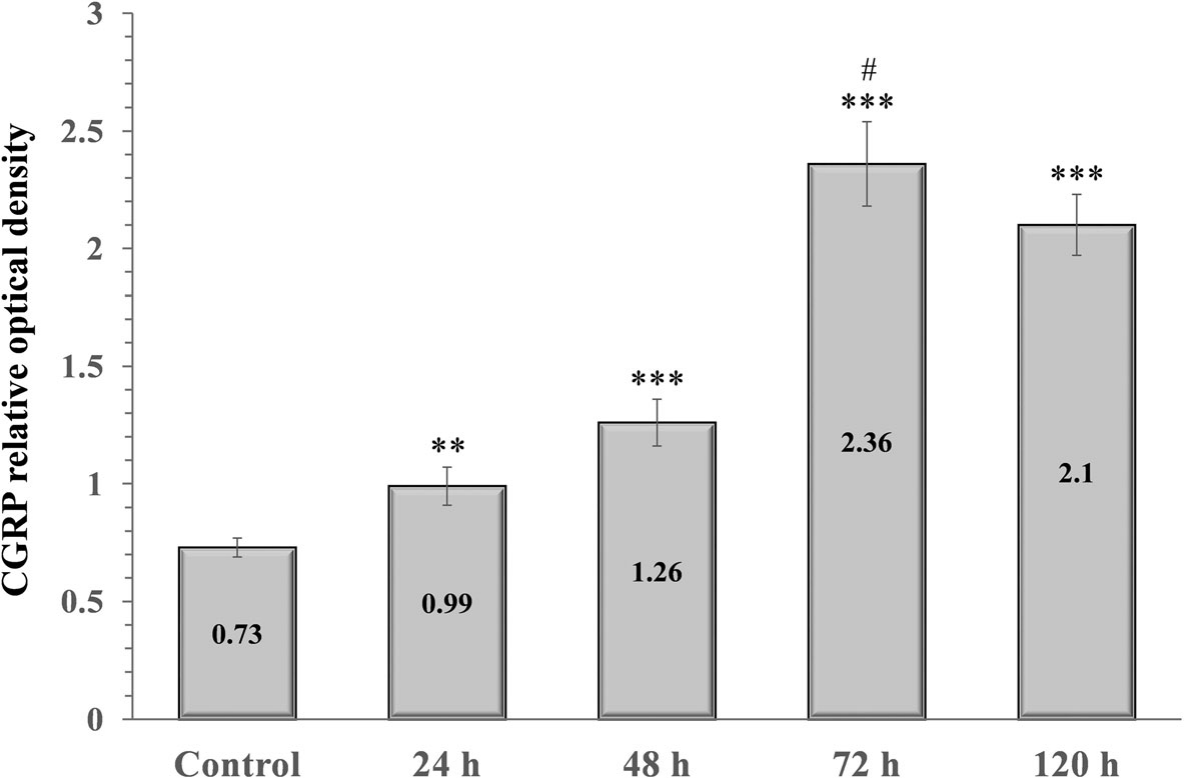
Relative optical density of the CGRP protein in the TNC following orofacial CFA treatment. *** *p* < 0.001 vs. Control group, ** *p* < 0.01 vs. Control group, # *p* < 0.05 vs. 120 h group, Mean ± SD, *n* = 6

**Fig. 4:**
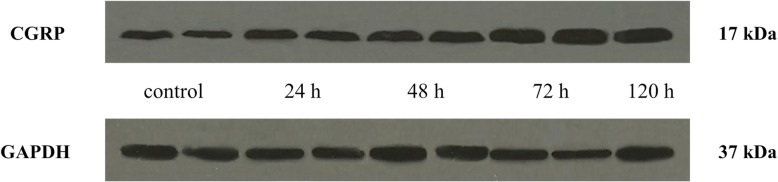
Western blot of CGRP and GAPDH expression in the TNC

CGRP expression in the TNC was elevated 24 (0.99), 48 (1.26), 72 (2.36) and 120 (2.1) hours after CFA injection compared to the control group (0.73). The highest CGRP concentration was detected 72 h after CFA treatment. The difference was significant between the 72-h and 120-h groups, therefore we did not feel it was necessary to include other group investigations (Figs. 3 and 4).

### Orofacial CFA injection decreased the mechanonociceptive threshold

**Fig. 5:**
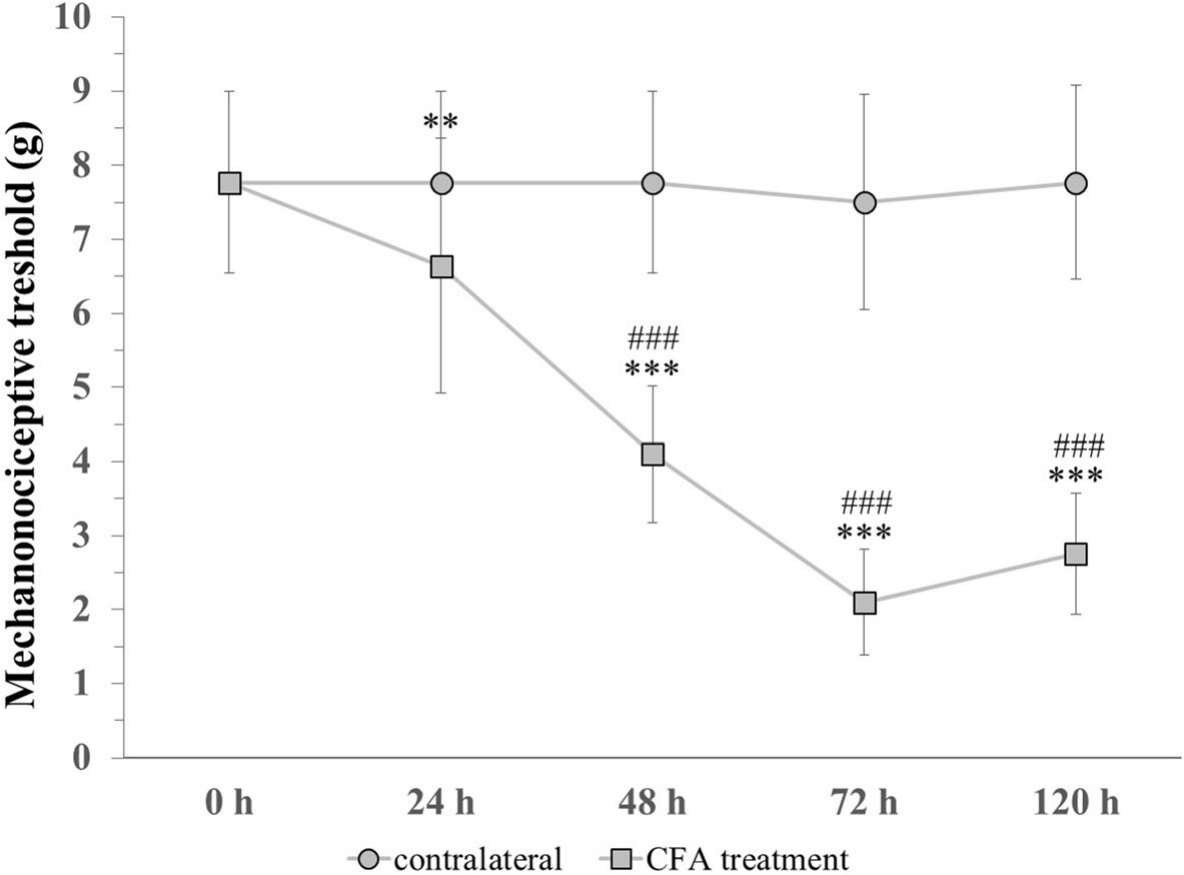
Changes in mechanical theshold before (0) and 24, 48, 72, 120 h after CFA treatment. *** *p* < 0.001 vs. Control measurement (0 h) in CFA treatment group, ** *p* < 0.01 vs. Control measurement (0 h) in CFA treatment group, ### *p* < 0.001 vs. contralateral side. Mean ± SD, *n* = 6–24

The facial mechanonociceptive threshold decreased significantly compared to the contralateral side 48, 72 and 120 h after CFA treatment. Compared to the control measurement (7.77), the threshold was reduced significantly 24 (6.64), 48 (4.1), 72 (2.1) and 120 (2.77) hours after CFA treatment. Allodynia reached its maximum at 72 h, as the threshold change was lower by 120 h. We did not find significant differences in the threshold of the contralateral whisker pad area (Fig. 5).

### Correlation between neuropeptides expression and mechanical hyperalgesia

**Fig. 6:**
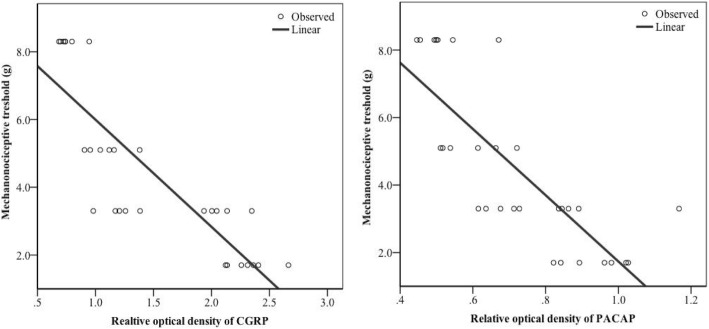
Negative, linear correlation between expression levels of neuropeptides and mechanonociceptive threshold

Reverse relationship was observed between the concentrations of neuropeptides and the value of the evoked mechanical threshold (CFA treated whisker pad) depending on the time. All data of the saline treated control group and the CFA treated groups were involved in the statistical probe. Regression analyses have revealed that negative, linear correlation is found between the expression levels of preproPACAP or CGRP and the levels of hyperalgesia. Then data were mixed with bootstrap analyses (1000x), which resulted in the following values: *n* = 30, p_CGRP_ < 0.001, R_CGRP_ = -0.846; *n* = 30, p_preproPACAP_ < 0.001, R_preproPACAP_ = − 0.792 (Fig. 6).

## Discussion

Migraine research in recent years has focused on neuropeptides as potential pathogenic factors and possible therapeutic alternatives. Neuropeptides such as hemokinin A, neurotensin, substance P (SP), CGRP and PACAP1–38 can activate mast cells, leading to the secretion of vasoactive, proinflammatory and neuro-sensitizing mediators, thereby contributing to the activation of TS [26, 27].

The aim of the present study was to investigate the simultaneous expression of CGRP and preproPACAP in the central region of the TS in a time-dependent manner following orofacial CFA treatment. Orofacial CFA injection evokes significant CGRP and preproPACAP increase in the TNC 24 h after treatment. The neuropeptide levels reach a maximum at 72 h after CFA injection, corresponding to the peak of facial allodynia.

Our opinion is that the CFA-induced inflammation can evoke continuous and long-term neuropeptide release in the TNC, which is accompanied by manifestation of mechanical hyperalgesia. It is assumed that the CFA can maintain this effect until 72 h, then both the preproPACAP and CGRP level start to decrease in the absence of stimulus, which causes the relief of allodynia. Our presumption is supported by a recent study, where the repetitive electrical stimulation of dura mater induced elevation in the expression level of CGRP and PACAP in the TRG and TNC in rat depending on the stimulation periods (1, 3 and 7 days). It suggests that the number of stimulations can influence the release and effects of neuropeptides [28]. Our results are consistent with a previous study, where the orofacial CFA injection provoked increased CGRP expression in the TNC, which elevation reached its maximum 3 days after CFA treatment [12]. In another study, activated microglial cells were detected in the ipsilateral TNC and in the cervical dorsal horn 72 h after orofacial CFA treatment of rats [29]. In this context, the activated microglial cells might also be involved to the mechanism of central sensitization and nociception [30–32]. It is proved that an antagonist of P2X4 microglia receptor blocked the NTG-induced c-Fos and CGRP release in the TNC, subsequently the hyperalgesia [33]. Results of our functional test showed that orofacial CFA injection can already cause a significant increase in mechanical allodynia 24 h after treatment. Allodynia reached its maximum on day 3, as the threshold change was lower by day 5. These results are consistent with observations from animal investigations where the CFA induced chronic pain-like behaviour 3 days after treatment [12, 14, 34]. Additionally, it was also demonstrated that chronic stimulation of dura mater can elicit facial cutaneous allodynia during the migraine chronification, via CGRP and PACAP elevation. One of the potential mechanisms, which can mediate the effect of PACAP is that its autoreceptor (PAC1) can influence the G-protein-coupled downstream effects via regulating the pre- and postsynaptic events [28]. Nevertheless, the moderating functional alterations might also be explained by the desensitization of receptors, but further investigations are needed to answer this hypothesis.

Another important result of present study is that the alterations of CGRP and preproPACAP expression show correlation with change of mechanical threshold. Several studies have proved to the key role of CGRP and PACAP1–38 mediators of neurogenic inflammation and modulators of pain inputs [2, 35, 36]. The release of these neuropeptides in the central nervous system facilitate nociceptive signalling and in the periphery contribute to vasodilatation of meningeal vessels and neurogenic inflammation [37]. Based on our results probable that the CFA-induced CGRP and PACAP1–38 increase play crucial role in triggering central sensitization, thereby regulation of these peptides may influence the rate of mechanical hyperalgesia [38].

### Role of neuropeptides in preclinical migraine models

A classical study confirmed that the concentration of CGRP and SP were increased during electrical stimulation of TRG (ES-TRG) of the external jugular vein of cats [39]. A former study suggests that ES-TRG resulted in significantly elevated PACAP1–38 immunoreactivity 180 min after ES-TRG of the plasma and PACAP1–38 and PACAP1–27 immunoreactivity in the TNC. Besides ES-TRG, the intraperitoneal administration of nitroglycerin (NTG) also induced an increase in PACAP1–38 and PACAP1–27 expression 3 h after the treatment in the TNC [3]. A study showed that PACAP1–38 administration can result in increased CGRP expression in the TNC, which points to a potential connection between release of CGRP and PACAP1–38 [40]. Co-expression of CGRP and PACAP1–38 was investigated: 23% of the neurons expressed both CGRP and PACAP1–38 in rat TRG, and CGRP (49%) was expressed in more neuronal somas compared to PACAP1–38 (29%) [41]. In a preclinical model of migraine, the simultaneous release of CGRP and PACAP was detected: a chronic NTG treatment caused increased concentrations of these peptides in the plasma of rats, furthermore the intervention evoked mechanical and thermal hyperalgesia [38].

Nevertheless the activation of TS could be formed by different CFA treatments, which results pain associated pathological states, including migraine, neuralgias and TMJ disorders [25]. In our previous study, we investigated the effect of CFA on the expression of mitogen-activated protein kinases (MAPK), which play essential roles in pain processing. Administration of CFA in the TMJ caused significant extracellular signal-regulated kinase 1/2 (ERK1/2) and p38 MAPK increase in the TRG [42]. Dural administration of CFA resulted elevated ERK1/2, interleukin-1β and CGRP expression in the TRG [11], as well as increased c-fos and glutamate immunoreactivity in the TNC and cervical neurons [43]. Moreover, gene expression alterations (CGRP, Iba1, GFAP, etc.) were detected following orofacial CFA treatment in the TRG and TNC [12], which suggest that the CFA induced neuroinflammation can evoke increased CGRP and PACAP1–38 levels.

### Role of neuropeptides in clinical studies

In migraineurs, the level of CGRP in the peripheral blood increases during a migraine attack as compared to the attack free [8, 39, 44, 45]. A very similar observation has recently been made for PACAP1–38 as well, suggesting a potential biomarker function of PACAP1–38 in the disease [8]. In addition, similarly to CGRP [35], intravenous administration of PACAP1–38 induced headache and vasodilatation, both in healthy subjects and patients suffering from migraine, whereas it delayed migraine-like attacks only in migraineurs [46, 47]. Magnetic resonance imaging angiography examinations proved that PACAP1–38 induced headache is associated with prolonged vasodilatation of the middle meningeal artery (MMA), but not the middle cerebral artery (MCA). Sumatriptan was able to alleviate the headache, which mirrored the contraction of the MMA, but not the MCA, suggesting that PACAP1–38-induced headaches may arise from the extracerebral arteries [48]. Correlation was showed between the interictal plasma PACAP1–38 immunoreactivity and the microstructural integrity of the white matter in migraineurs [5]. These data support the idea that neuropeptides could be a good candidate for the new therapeutic approaches.

Nowadays the therapies based on monoclonal antibodies of CGRP seem to be promising in the prevention of migraine [49]. Recently, a phase 3 clinical trial showed that 12 months of treatment with galcanezumab, which is a fully humanized CGRP monoclonal antibody, was safe and associated with a reduction in the number of monthly migraine headache days [50]. Besides galcanezumab, erenumab, eptinezumab and fremanezumab were able to reduce the frequency of attacks in patients with episodic migraine [51].

Considering the similar behaviours of CGRP and PACAP1–38, the therapies aimed at PACAP1–38 may also be useful for those who have an inadequate response to therapeutics directed at CGRP or its receptors. A phase 2a, randomized, double blind, placebo-controlled study is underway to appraise the efficacy and safety of a PAC1 receptor antibody (AMG 301) in subjects with chronic or episodic migraine *(Study to Evaluate the Efficacy and Safety of AMG 301 in Migraine Prevention.* *https://clinicaltrials.gov/ct2/show/NCT03238781**. Accessed 02 May 2018)*. Preclinical studies are also evaluating a monoclonal antibody (ALD1910) targeting PACAP1–38 *(ALD1910 – migraine prevention.* *alderbio.com**.* *https://www.alderbio.com/pipeline/ald1910/**. Accessed 19 May 2018,* [10]. Monoclonal antibodies may be crucial in the therapy of migraine however it needs further examinations to certify their relevance.

## Conclusion

Our results provided the first direct evidence that the expression levels of CGRP and preproPACAP simultaneously increase after CFA induced trigeminal activation in the central region of the TS. Correlations, which were found between the alterations of CGRP/preproPACAP expression and mechanical threshold prove the influence of neuropeptides in the mechanism of hyperalgesia. Data of the present study contribute to the better understanding of migraine pathogenesis and support the idea that neuropeptides may have therapeutic value in migraine treatment.

## Abbreviations

BSA: Bovine serum albumin
CFA: Complete Freund’s adjuvant
CGRP: Calcitonin gene-related peptide
EDTA: Ethylenediaminetetraacetic acid
ERK1/2: Extracellular signal-regulated kinase 1/2
ES-TRG: Electrical stimulation of the TRG
MAPK: Mitogen-activated protein kinase
MCA: Middle cerebral artery
MMA: Middle meningeal artery
NTG: Nitroglycerin
PACAP: Pituitary adenylate cyclase-activating polypeptide
PACAP1–27: 27 amino acid form of PACAP
PACAP1–38: 38 amino acid form of PACAP
PBS: Phosphate-buffered saline
SDS: Sodium dodecyl sulfate
SP: Substance P
TBST: Tris-buffered saline containing Tween 20
TMJ: Temporomandibular joint
TNC: Trigeminal nucleus caudalis
TRG: Trigeminal ganglion
TS: Trigeminovascular system
VIP: Vasoactive intestinal polypeptide
